# Identification of two genes associated with recurrence in Paget’s disease and construction of a predictive model

**DOI:** 10.3389/fgene.2026.1784429

**Published:** 2026-05-13

**Authors:** Ying Chen, Yang Chen, Yan Wan, Wenhui Guo, Bingying Huang, Qinqin Long, Yu He, Pengfei Cha

**Affiliations:** 1 Department of Plastic Surgery, Dermatology Hospital of Fuzhou, Fuzhou, China; 2 Department of Breast Surgery, Fujian Medical University Union Hospital, Fuzhou, China; 3 The Department of Presbyatrics, 900th Hospital of PLA Joint Logistic Support Force, Fuzhou, China; 4 Fuzhou Dermatology Hospital, Fuzhou, China

**Keywords:** KLF13, Paget’s disease, prognosis, TIA1, weighted gene co-expression network analysis

## Abstract

**Background:**

Mammary Paget’s disease (MPD) and extramammary Paget’s disease (EMPD) exhibit distinct clinical behaviors, yet the underlying molecular drivers of prognosis remain poorly characterized. This study aimed to identify key prognostic genes and construct a predictive model for Paget’s disease (PD).

**Methods:**

RNA sequencing was performed on MPD and EMPD tissues. Hub genes were screened using weighted gene co-expression network analysis (WGCNA). Their prognostic value was validated via time dependent receiver operating characteristic (ROC) curves, Kaplan-Meier survival analysis, and Decision curve analysis (DCA) in internal and external cohorts. A risk-score model was subsequently developed based on the identified genes.

**Results:**

RNA-seq analysis revealed distinct functional profiles between MPD and EMPD, with recurrence in MPD associated with developmental and differentiation pathways, while EMPD recurrence was linked to immune and inflammatory processes. WGCNA identified KLF13 and TIA1 as hub genes. In the internal cohort, both genes were significantly overexpressed in patients with recurrence (KLF13: 22.74 ± 3.41 vs. 15.36 ± 4.91, P < 0.001; TIA1: 11.69 ± 2.48 vs. 7.74 ± 1.62, P < 0.001). And the KLF13 and TIA1 were also validated by qPCR in the internal cohort. The genes also demonstrated prognostic validity in an independent Chinese PD cohort. A risk-score model incorporating KLF13 and TIA1 effectively stratified patients into high- and low-risk groups with distinct outcomes in both internal and external validation sets. Moreover, we knocked down TIA1 expression in MDA-MB-231 cells, and both *in vitro* and *in vivo* results demonstrated that TIA1 functions as an oncogene.

**Conclusion:**

KLF13 and TIA1 are robust prognostic biomarkers in PD. The developed risk-score model provides a valuable tool for predicting recurrence and personalizing patient management. In addition, both *in vitro* and *in vivo* findings confirmed that TIA1 functions as an oncogene.

## 1 Introduction

Paget’s disease (PD) is a malignant cutaneous neoplasm that resembles eczema, hence it is also referred to as eczematoid carcinoma. The histopathological hallmark of cutaneous PD is the presence of Paget cells within the epidermis. Clinically, based on the site of occurrence, PD can be classified into mammary Paget’s disease (MPD) and extramammary Paget’s disease (EMPD). MPD accounts for 1%–3% of primary breast tumors and is often associated with ductal carcinoma *in situ* or invasive ductal carcinoma. It frequently originates in the complex region of the nipple/areola and spreads to the surrounding skin (Duan et al., 2012). Zhao et al. reported that among 3,431 MPD patients, 2,962 presented with ductal carcinoma *in situ* or invasive ductal carcinoma (Zhao et al., 2018). Although EMPD can occur in any cutaneous or mucosal site, it is most commonly found in the anogenital region (Lloyd and Flanagan, 2000). EMPD can be divided into primary and secondary forms, with secondary EMPD typically involving cutaneous metastases from other malignancies, such as colorectal, bladder, endometrial, and gastric cancers (Konstantinova et al., 2016).

Although both MPD and EMPD are considered PD, whether they represent the same disease entity continues to be debated. Some studies suggest that EMPD and MPD share common biomarkers. The prevailing view is that in MPD, Paget cells originate from mammary ductal carcinoma cells that migrate through the basement membrane to the nipple (Hu et al., 2022; Chen et al., 2019), whereas EMPD is thought to arise from apocrine glands (Song et al., 2020; Lopes Filho et al., 2015). MPD is diagnosed based on a combination of clinical, imaging, and histopathological findings. Its treatment strategy is primarily determined by postoperative pathology—specifically, the presence or absence of associated breast cancer and lymph node metastasis. In contrast, EMPD is mainly managed with local surgical excision supplemented by radiotherapy, depending on the site of involvement. Numerous studies have compared samples from PD patients with normal skin samples in an attempt to identify disease-specific biomarkers. However, few studies have investigated potential biomarker differences between MPD and EMPD. Additionally, while treatment approaches for MPD and EMPD remain debated, radical surgery is currently regarded as the primary treatment modality for both (Lloyd and Flanagan, 2000; Lopes Filho et al., 2015; Bunker et al., 1992). Whether adjuvant therapy is required postoperatively remains unclear. A limited number of studies indicate that EMPD can also occur on breast skin (Fern et al., 2018), highlighting the importance of accurately distinguishing between MPD and EMPD to guide subsequent therapy. Hence, elucidating the differences in molecular markers between MPD and EMPD and delineating their corresponding molecular mechanisms will be of substantial value in guiding clinical decision-making for subsequent treatments.

In this context, this study aimed to investigate the functional differences between MPD and EMPD using WGCNA, pinpoint relevant hub genes for PD patients. Subsequently, these candidate hub genes were validated using patient tissue samples, along with internal and external validation datasets.

## 2 Materials and methods

### 2.1 Subjects and collection

A total of 11 patients with EMPD and 28 patients with MPD were enrolled in this study. EMPD cases were recruited from the Dermatology Hospital of Fuzhou (January 2019 to December 2021), while MPD cases were obtained from the Fujian Medical University Union Hospital (January 2020 to January 2021). Tumor samples from all patients underwent RNA sequencing and qPCR. Clinicopathological characteristics are summarized in Supplementary Table S1. All participants provided written informed consent voluntarily. Inclusion criteria were: (1) primary tumor resection with R0 margins and no prior treatment; (2) pathological confirmation of Paget cells within the tumor; (3) age between 18 and 80 years; and (4) EMPD tumor originating from the perineal region, specifically the external genitalia, anus, and perineal skin. Patients were excluded if they: (1) had a history of or concurrent other malignancies, or (2) presented with distant metastasis at diagnosis.

### 2.2 RNA extraction, enrichment, reverse transcription, and quality control

Total RNA was isolated using TRIzol® reagent (Thermo Fisher Scientific), followed by purification with RNeasy Mini Kit (Qiagen). Polyadenylated mRNA was enriched by binding to oligo (dT)-coated magnetic beads (Thermo Fisher Scientific) through hybridization to the poly(A) tail, ensuring high-quality mRNA selection for downstream analysis. mRNA was reverse transcribed, amplified, and quality control which were performed by the Sangon Biotech Inc (Shanghai, China). Libraries with insert sizes of 300–500 bp and concentrations ≥10 nM were selected for sequencing.

### 2.3 Sequencing and data analysis

Total RNA was extracted using TRIzol® reagent (Thermo Fisher Scientific, United States) and purified with the RNeasy Mini Kit (Qiagen, Germany). Polyadenylated mRNA was subsequently enriched by hybridization of the poly(A) tail to oligo (dT)-coupled magnetic beads (Thermo Fisher Scientific). All subsequent steps, including reverse transcription, amplification and quality control, were conducted by Sangon Biotech Inc. (Shanghai, China). Finally, libraries with an insert size of 300–500 bp and a concentration ≥10 nM were prepared for sequencing.

Paired-end sequencing was conducted on an Illumina HiSeq™ 4000 platform (Illumina, Inc., United States). Raw sequencing data were generated in FASTQ format for each sample. Initial quality assessment was performed using FastQC. Adapter contamination and low-quality bases were subsequently removed using Trimmomatic (v0.39) with the following parameters: SLIDINGWINDOW:4:20 MINLEN:36. Cleaned reads were aligned to the human reference genome (GRCh38/hg38) using HISAT2 under default settings. Transcript assembly and gene-level abundance estimation were carried out with StringTie based on Ensembl annotations. Differentially expressed mRNAs (DEMs) were identified using a threshold of |log_2_ (fold change)| ≥ 1. Functional interpretation of the DEMs was performed through Gene Ontology (GO) enrichment analysis and Kyoto Encyclopedia of Genes and Genomes (KEGG) pathway analysis, employing a standard hypergeometric test.

### 2.4 Data collection and immunohistochemical analysis

To validate the expression of KLF13 and TIA1 in PD tissues, consecutive patients with EMPD who underwent radical resection between 2017 and 2021 at the Dermatology Hospital of Fuzhou, as well as patients with MPD who underwent radical resection between 2017 and 2020, were included for immunohistochemical (IHC) analysis. A total of 38 EMPD and 56 MPD patients were enrolled in this validation cohort. The patients inclusion criteria were: (1) primary tumor resection with R0 margins and no prior treatment; (2) pathological confirmation of Paget cells within the tumor; (3) age between 18 and 80 years; and (4) EMPD tumor originating from the perineal region, specifically the external genitalia, anus, and perineal skin. Patients were excluded if they: (1) had a history of or concurrent other malignancies, or (2) presented with distant metastasis at diagnosis. The study was approved by the Institutional Review Board of the Dermatology Hospital of Fuzhou (approval number: FZSP-LL- KYXM- 02).

Protein expression of KLF13 and TIA1 was evaluated using the streptavidin-biotin complex IHC method. Immunohistochemical staining for KLF13 (catalog no. DF13113, Affinity Biosciences, polyclonal, 1:100) and TIA1 (catalog no. DF12176, Affinity Biosciences, polyclonal, 1:100) was performed on surgical tissue sections. Negative controls were established by substituting the primary antibody with phosphate-buffered saline (PBS), while positive control images were sourced from GE Healthcare Life Sciences. Immunoreactivity was subsequently evaluated using a semi-quantitative approach. For each specimen, five non-overlapping fields at ×400 magnification were systematically selected from predefined regions (upper, central, lower, left, and right). Staining intensity was graded on a scale of 0–3 (0, no staining; 1, light yellow; 2, brown; 3, dark brown), and the proportion of positively stained cells was scored as 0 (<5%), 1 (5%–25%), 2 (25%–50%), 3 (50%–75%), or 4 (>75%). An H-score for each field was then derived by multiplying the intensity and percentage scores. The final H-score for each case was determined as the mean of the scores from all five fields. Based on this score, protein expression levels were categorized as low (H-score<4) or high (H-score≥4). All IHC assessments were conducted in a double-blind manner.

### 2.5 Co-expression network construction and hub gene identification

The co-expression network analysis was performed using the WGCNA package in R (Zhang and Horvath, 2005; Horvath and Dong, 2008; Mason et al., 2009). Briefly, a signed correlation matrix was constructed, and an appropriate soft-thresholding power was selected to achieve scale-free topology. A topological overlap matrix (TOM) was then generated to assess network interconnectedness (Botía et al., 2017; Yip and Horvath, 2007; Ivanova et al., 2013). Modules were identified using hierarchical clustering and dynamic tree cutting (Supplementary Figure S1). Based on the recurrence status, gene significance for recurrence was calculated, and module-trait associations were evaluated. The cyan module showed the strongest correlation with recurrence and was therefore selected for further analysis. Within this module, genes with high intramodular connectivity and high gene significance were considered candidate hub genes. To further refine the selection, least absolute shrinkage and selection operator (LASSO) regression was applied, which identified KLF13 and TIA1 as the most predictive hub genes associated with recurrence.

### 2.6 ssGSEA and CIBERSORT analysis

To assess immune-related gene set activity in individual PD samples, we performed single-sample gene set enrichment analysis (ssGSEA) on a predefined panel of 29 immune signatures. For tumor microenvironment characterization, we applied two complementary computational methods. CIBERSORT was used to estimate the relative abundances of 22 tumor-infiltrating immune cell types. In parallel, the ESTIMATE algorithm provided immune and stromal scores, along with an estimate of tumor purity, for each sample.

### 2.7 Cell culture and lentiviral-mediated TIA1 knockdown

The human breast cancer cell line MDA-MB-231 was procured from the National Infrastructure of Cell Line Resource (China). To generate TIA1 knockdown (TIA1-KD) cells, short hairpin RNA (shRNA) oligonucleotides targeting TIA1 (Invitrogen) were synthesized and cloned into the pLKO.1-TRC vector via AgeI and EcoRI restriction sites. For lentivirus production, HEK293T cells were co-transfected with 5 μg of the pLKO.1-puro construct containing the shRNA sequence, along with 5 μg of packaging plasmid and envelope vectors, using Lipofectamine 2000 (Invitrogen) according to the manufacturer’s specifications. Viral supernatants were collected 48 h post-transfection. MDA-MB-231 cells were subsequently transduced with lentivirus expressing either TIA1-targeting shRNA or control shRNA (Control) for 24 h. Following a 48-h recovery period, successfully transduced cells were selected with puromycin (4 μg/mL; Sigma-Aldrich) for an additional 48 h prior to downstream functional analyses.

### 2.8 Colony formation and cell proliferation assays

For colony formation assessment, cells were seeded into 6-well plates at a density of 500 cells per well and maintained in culture for 7 days. Formed colonies were fixed with 4% paraformaldehyde (15 min), stained with 1% crystal violet (10 min), and those exceeding 1.5 mm in diameter were enumerated under a microscope. Cell proliferation was evaluated using the Cell Counting Kit-8 (CCK-8; Dojindo Laboratories) as per the manufacturer’s protocol. Absorbance was measured at 450 nm using a microplate reader, and cell viability was expressed relative to control groups.

### 2.9 Reverse transcription-quantitative PCR (RT-qPCR)

Total RNA was isolated from cultured cells and patient tissue specimens using TRIzol reagent (Invitrogen; Thermo Fisher Scientific) following the manufacturer’s instructions. One microgram of RNA was reverse-transcribed into cDNA using M-MLV Reverse Transcriptase (Promega Corporation). Quantitative PCR was subsequently performed on an ABI 7500 Real-Time PCR System (Thermo Fisher Scientific) employing gene-specific primers (listed in Supplementary Table S2).

### 2.10 Tumor xenografts in the Rat

A total of six male athymic nude mice (15–20 g, 6–8 weeks old) were obtained from Shanghai SLAC Laboratory Animal Co., Ltd (China). All animals were cared for and treated in accordance with institutional guidelines. Tumor xenografts were established by subcutaneous injection of 200 μL of cell suspension (1 × 10^7^ cells/mL) into each foreleg of the nude mice. The cells used were MDA-MB-231 for the control group (right foreleg) and MDA-MB-231 with TIA1 knockdown for the experimental group (left foreleg). Tumor size was assessed weekly by measuring the longest diameter, and measurements were continued for 4 weeks. In addition, fluorescence detection was performed at the first and fourth weeks. All experimental procedures were approved by the Committee of Fujian Medical University.

### 2.11 Statistical analysis

Statistical analyses were conducted using SPSS (version 27.0), GraphPad Prism 9, and R (version 4.4.3). Continuous variables were expressed as mean ± standard deviation and compared using analysis of variance. Survival outcomes were evaluated by the Kaplan-Meier method with log-rank tests. The optimal cutoff values for hub gene expression were determined using the X-tile software. A prognostic risk-score model was constructed based on Cox proportional hazards regression, and its clinical utility was assessed using Decision curve analysis (DCA) for disease-free survival. The relationship between hub genes and tumor-infiltrating immune cells was explored via the TIMER database. A p-value <0.05 was considered statistically significant.

## 3 Results

### 3.1 Cluster analysis, GO enrichment and KEGG analysis in PD patients

**FIGURE 1:**
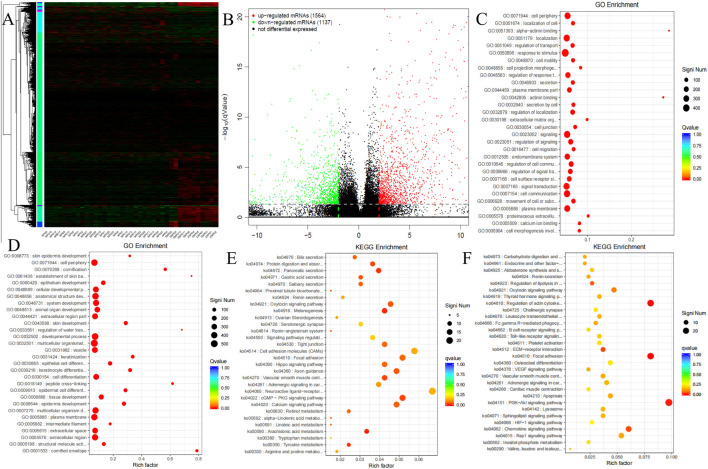
mRNAs expression profile comparison between MPD and EMPD patients. Gene Ontology (GO) functional and Kyoto Encyclopedia of Genes and Genomes (KEGG) pathway analysis of the differentially expressed genes. **(A)** The hierarchical clustering of all targets value of mRNA expression profiling among samples. **(B)** between the MPD and EMPD group. The purple dots indicated the upregulated genes of mRNAs and the green dots indicated the downregulated genes of mRNAs. **(C)** GO functional analysis of the top ten functional classifications of the upregulated genes. **(D)** GO functional analysis of the top ten functional classifications of the downregulated genes. **(E)** KEGG pathway analysis of the top ten significant pathways of upregulated genes. **(F)** KEGG pathway analysis of the top ten pathways of downregulated genes. MPD: Mammary Paget’s disease, EMPD: extramammary Paget’s disease.

To elucidate the molecular underpinnings of MPD and EMPD patients, we performed transcriptome profiling to compare mRNA expression between EMPD patients (N = 11) and MPD patients (N = 28). A supervised hierarchical cluster analysis of gene expression profiling data showed that the 2 groups had a clustering trend (Figure 1A). Volcano plot analysis (Figure 1B) identified a total of 2701 differentially expressed genes (DEGs) with statistical significance (adjusted P-value <0.05, |log2 fold change| > 2), comprising 1564 upregulated (purple dots) and 1137 downregulated (green dots) genes in the MPD group compared to the EMPD group.

GO enrichment analysis was performed to investigate the molecular mechanism of differently expressed genes involved in PD patients. We detected the top 10 significant GO terms enriched in both the significantly upregulated and downregulated genes in PD patients, respectively (Figures 1C,D). The results showed that the top 3 significant GO terms were related to the epidermis development, skin development, and cornification in upregulated genes. In the downregulated genes, the top three significant GO terms were related to the cell periphery, plasma membrane, and regulation of localization. Additionally, we analyzed the differential genes in PD patients by KEGG analysis (Figures 1E,F). The results demonstrated that the top three KEGG pathways were related to the Arachidonic acid metabolism, Tyrosine metabolism, and Melanogenesis in upregulated genes. The top three downregulated genes were the Focal adhesion, Regulation of actin cytoskeleton, and PI3K-Akt signaling pathway.

### 3.2 Cluster analysis, GO enrichment and KEGG analysis in recurrence and non-recurrence patients

**FIGURE 2:**
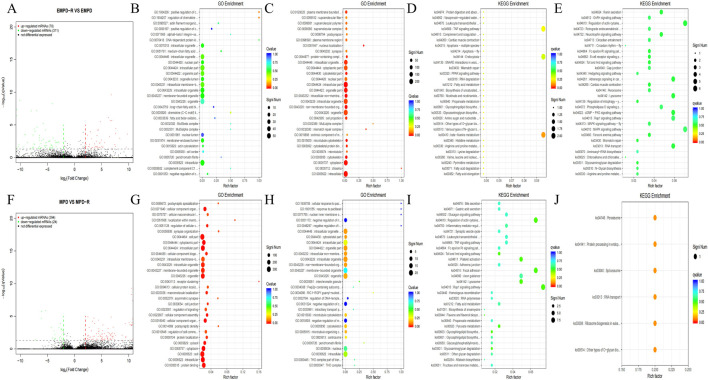
mRNAs expression profile comparison between recurrence and non-recurrence in the EMPD and MPD patients, respectively. Gene Ontology (GO) functional and Kyoto Encyclopedia of Genes and Genomes (KEGG) pathway analysis of the differentially expressed genes. **(A)** between the recurrence and non-recurrence group in the EMPD patients. The purple dots indicated the upregulated genes of mRNAs and the green dots indicated the downregulated genes of mRNAs. **(B)** GO functional analysis of the top ten functional classifications of the upregulated genes. **(C)** GO functional analysis of the top ten functional classifications of the downregulated genes. **(D)** KEGG pathway analysis of the top ten significant pathways of upregulated genes. **(E)** KEGG pathway analysis of the top ten pathways of downregulated genes. **(F)** between the recurrence and non-recurrence group in the MPD patients. The purple dots indicated the upregulated genes of mRNAs and the green dots indicated the downregulated genes of mRNAs. **(G)** GO functional analysis of the top ten functional classifications of the upregulated genes. **(H)** GO functional analysis of the top ten functional classifications of the downregulated genes. **(I)** KEGG pathway analysis of the top ten significant pathways of upregulated genes. **(J)** KEGG pathway analysis of the top ten pathways of downregulated genes. MPD: Mammary Paget’s disease, EMPD: extramammary Paget’s disease.

To elucidate the molecular underpinnings of recurrence and non-recurrence in PD patients, we performed transcriptome profiling to compare mRNA expression between recurrence and non-recurrence patients in MPD and EMPD, respectively. Volcano plot analysis (Figure 2A) identified a total of 318 DEGs with statistical significance in the recurrence group compared to the non-recurrence group in the EMPD patients. GO enrichment analysis was performed to investigate the molecular mechanism of differently expressed genes involved in recurrence and non-recurrence patients in EMPD patients. We detected the top 10 significant GO terms enriched in both the significantly upregulated and downregulated genes in recurrence and non-recurrence patients in EMPD patients (Figures 2B,C). The results showed that the top 3 significant GO terms were related to the regulation of chemokine (C-C motif) ligand 2 secretion, intracellular organelle part, and organelle part in upregulated genes. In the downregulated genes, the top three significant GO terms were related to the non-membrane-bounded organelle, intracellular part, and cytoskeleton. Additionally, we analyzed the differential genes in recurrence and non-recurrence patients in EMPD patients by KEGG analysis (Figures 2D,E). The results demonstrated that the top three KEGG pathways were related to the beta-Alanine metabolism, Glycosaminoglycan biosynthesis - keratan sulfate, and TNF signaling pathway in upregulated genes. The top three downregulated genes were the Renin secretion, Toll and Imd signaling pathway, and Glycosaminoglycan degradation.

Volcano plot analysis (Figure 2F) identifieda total of 418 DEGs with statistical significance in the recurrence group compared to the non-recurrence group in the MPD patients. GO enrichment analysis was performed to investigate the molecular mechanism of differently expressed genes involved in recurrence and non-recurrence patients in MPD patients. We detected the top 10 significant GO terms enriched in both the significantly upregulated and downregulated genes in recurrence and non-recurrence patients in MPD patients (Figures 2G,H). The results showed that the top 3 significant GO terms were related to the cytoplasmic part, intracellular, andcellular component organization in upregulated genes. In the downregulated genes, the top three significant GO terms were related to the intracellular organelle, intracellular organelle part, and microtubule cytoskeleton. Additionally, we analyzed the differential genes in recurrence and non-recurrence patients in MPD patients by KEGG analysis (Figures 2I,J). The results demonstrated that the top three KEGG pathways were related to the Adrenergic signaling in cardiomyocytes, Oxytocin signaling pathway, and Endocrine and other factor-regulated calcium reabsorption in upregulated genes. The top three downregulated genes were the Other types of O-glycan biosynthesis, Peroxisome, and Ribosome biogenesis in eukaryotes.

### 3.3 Identification of hub genes via WGCNA and LASSO regression

**FIGURE 3:**
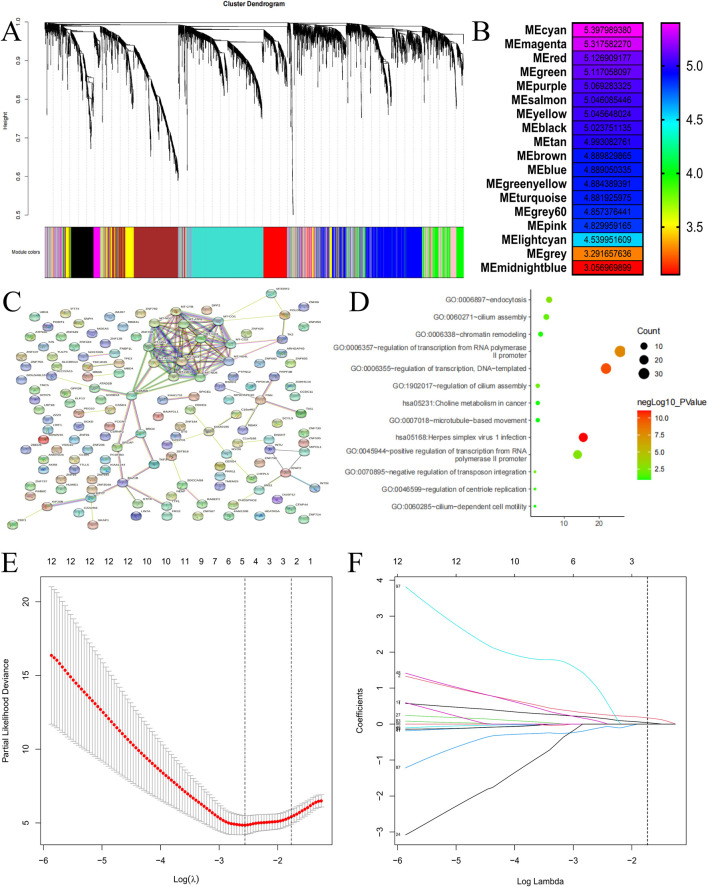
Weighted gene co-expression network analysis (WGCNA) and hub gene screened. **(A)** Dendrogram of genes based on a dissimilarity measure. **(B)** Heatmap of the correlation between all modules and recurrence. The scatter diagram of the relationship between the hub cyan module and recurrence. **(C)** Protein-protein interaction network of genes in the cyan module. **(D)** KEGG pathway and GO functional analysis of genes in cyan modules. **(E)** LASSO coefficient profiles of the 140 factors, **(F)** The AUC was estimated with cross-validation technique and the largest lambda value was chosen when the cross-validation error was within one standard error of the minimum. MPD: Mammary Paget’s disease, EMPD: extramammary Paget’s disease, AUC: Area under the curve, LASSO: Least absolute shrinkage and selection operator.

To identify the hub gene, we used the weighted co-expression network to analyze the RNA-seq (Figure 3A). A total of 19 modules were identified (Figure 3B), and the cyan module was the highest score which was defined as the most important module. Thus, the cyan module was selected as the hub modules for further analysis. A protein-protein interaction (PPI) network of genes within this cyan module was constructed (Figure 3C), and its functional analysis confirmed enrichment in regulation of transcription, DNA-templated and Choline metabolism in cancer which were based on the GO enrichment and KEGG analysis (Figure 3D). To refine the prognostic gene signature, we applied the Least Absolute Shrinkage and Selection Operator (LASSO) Cox regression analysis to the cyan module genes. The LASSO coefficient profiles are shown in Figure 3E. Through ten-fold cross-validation, we identified the optimal lambda value that minimized the cross-validation error (Figure 3F). This process ultimately selected two core hub genes: Kruppel-like factor 13 (KLF13) and T-cell intracellular antigen 1 (TIA1).

### 3.4 Hub gene identification and validation in the internal data set

**FIGURE 4:**
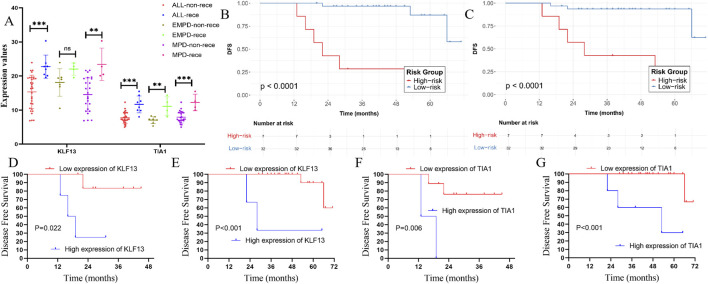
Validation of the hub genes in the internal data-set. **(A)** The hub genes expression in the recurrence and non-recurrence group in all patients, MPD, and EMPD, respectively (recurrence group vs. non-recurrence group in all patients: KLF13, 22.74 ± 3.41 vs. 15.36 ± 4.91, P < 0.001; TIA1, 11.69 ± 2.48 vs. 7.74 ± 1.62, P < 0.001; recurrence group vs. non-recurrence group in EMPD patients: KLF13, 22.05 ± 1.81 vs. 18.11 ± 4.04, P = 0.103; TIA1, 11.14 ± 2.77 vs. 7.09 ± 1.02, P < 0.01; recurrence group vs. non-recurrence group in MPD patients: KLF13, 23.42 ± 4.75 vs. 14.56 ± 4.92, P < 0.01; TIA1, 12.25 ± 2.42 vs. 7.92 ± 1.72, P < 0.001). **(B)** The K-M analysis of the KLF13 for the DFS in all patients in internal RNA-seq data-set; **(C)** The K-M analysis of the TIA1 for the DFS in the internal RNA-seq data-set; **(D)** The K-M analysis of the KLF13 for the DFS in EMPD in internal RNA-seq data-set; **(E)** The K-M analysis of the KLF13 for the DFS in MPD in internal RNA-seq data-set; **(F)** The K-M analysis of the TIA1 for the DFS in EMPD in internal RNA-seq data-set; **(G)** The K-M analysis of the TIA1 for the DFS in MPD in internal RNA-seq data-set. MPD: Mammary Paget’s disease, EMPD: extramammary Paget’s disease, K-M: Kaplan-Meier. *: <0.05, **: <0.01, ***: <0.001.

The prognostic significance of KLF13 and TIA1 was rigorously validated. In our internal RNA-seq dataset, both genes were significantly overexpressed in the recurrence group of all patients (KLF13: 22.74 ± 3.41 vs. 15.36 ± 4.91, P < 0.001; TIA1: 11.69 ± 2.48 vs. 7.74 ± 1.62, P < 0.001) and in the MPD subgroup (Figure 4A). The association was consistent but showed a non-significant trend for KLF13 in the EMPD subgroup. Critically, high expression of either KLF13 or TIA1 was a strong predictor of shorter disease-free survival (DFS). Kaplan-Meier (K-M) analysis in the internal cohort showed that patients with high KLF13 or TIA1 expression had significantly poorer DFS across all patients (Figures 4B,C) and in both MPD and EMPD subgroups (Figures 4D–G).

After a median follow-up period of 41 months (range, 13–71 months), and 8 patients experienced disease recurrence, with 4 cases each in the MPD and EMPD groups. Moreover, low expressions of KLF13 or TIA1 were associated with significantly better DFS compared to high expression [KLF13 low expression VS. high expression: 90.6% (3/32) vs.28.6% (5/7), P < 0.001; TIA1 low expression VS. high expression: 90.6% (3/32) vs.28.6% (5/7), P < 0.001], as demonstrated in Figures 4B,C). Moreover, in the MPD and EMPD patients, we found the similarly results that the high expression of KLF13 and TIA1 associated with the worse DFS, respectively. (Figures 4D–G).

### 3.5 Hub gene validation in external data set by the immunohistochemical

**FIGURE 5:**
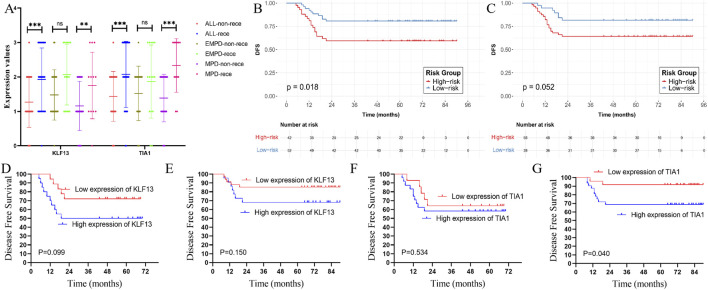
Validation of the hub genes in the external data-set. **(A)** The hub genes expression in the recurrence and non-recurrence group in all patients, MPD, and EMPD, respectively (recurrence group vs. non-recurrence group in all patients: KLF13, P < 0.001; TIA1, P < 0.001; recurrence group vs. non-recurrence group in EMPD patients: KLF13, P = 0.122; TIA1, P = 0.111; recurrence group vs. non-recurrence group in MPD patients: KLF13, P < 0.01; TIA1, P < 0.001). **(B)** The K-M analysis of the KLF13 for the DFS in all patients in external data-set; **(C)** The K-M analysis of the TIA1 for the DFS in the external data-set; **(D)** The K-M analysis of the KLF13 for the DFS in EMPD in external data-set; **(E)** The K-M analysis of the KLF13 for the DFS in MPD in external data-set; **(F)** The K-M analysis of the TIA1 for the DFS in EMPD in external data-set; **(G)** The K-M analysis of the TIA1 for the DFS in MPD in external data-set. MPD: Mammary Paget’s disease, EMPD: extramammary Paget’s disease. *: <0.05, **: <0.01, ***: <0.001.

To further verified the hub genes expression in the external data-set, we examined the expression level of KLF13 and TIA1 in the MPD and EMPD patients by IHC (Figure 5). The IHC analysis demonstrated that the hub genes protein were higher expression in the all recurrence group compared with the disease free group (P < 0.01) (Figure 5A). The grade of the KLF13, -, +, ++, +++, in the disease free group was 9, 33, 23, 2, respectively, and the grade -, +, ++, +++, in the recurrence group was 1, 9, 8, 9, respectively. The grade of the TIA1, -, +, ++, +++, in the disease free group was8, 23, 35, 1, respectively, and the grade -, +, ++, +++, in the recurrence group was 2, 5, 9, 11, respectively. Moreover, the similarly results that over-expression of the KLF13 and TIA1 were in the recurrence group compared with the disease free group were also found in the MPD and EMPD patients, respectively.

To further explore the hub genes’ predictive ability of prognosis. The K-M analysis was employed to analyze the prognosis in the PD patients. A total of 94 PD patients were enrolled in the present study consistence with 38 EMPD patients and 56 MPD patients. After a median follow-up period of 65 months (range, 5–90 months), and 15 cases of EMPD patients (15/38, 39.5%) and 12 cases of MPD patients (12/56, 21.4%) experienced recurrence. Moreover, low expressions of KLF13 or TIA1 were associated with significantly better DFS compared to high expression [KLF13 low expression VS. high expression: 80.8% (10/52) vs.59.5% (17/42), P < 0.001; TIA1 low expression VS. high expression: 81.6% (7/38) vs.64.3% (20/56), P < 0.001], as demonstrated in Figures 5B, 4C). Moreover, in the MPD and EMPD patients, we found the similarly results that the high expression of KLF13 and TIA1 associated with the worse DFS, respectively. (Figures 5D–G).

### 3.6 Hub genes, immune infiltration, and functional association

**FIGURE 6:**
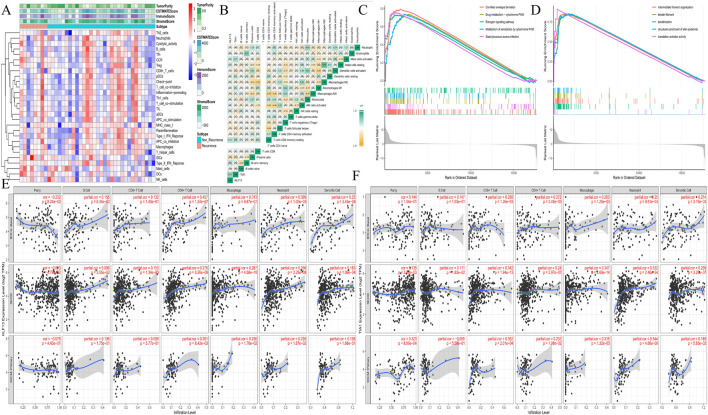
The function of the hub genes associated with the immunoinfiltration. **(A)** The heatmap of the recurrence or non-recurrence groups associated with the infiltration of immune cells and proportion of stroma in all patients. **(B)** The correction map of the hub genes expression and infiltration of immune cells. **(C)** The associated KEGG pathways of the recurrence. **(D)** The associated GO function of the recurrence. TIMER analysis the relationship between hub genes KLF13 **(E)**, TIA1 **(F)**, and tumor infiltrating immune cells in the BRCA-basal, SKCM, and SKCM-primary, receptively. GO: Gene Ontology, KEGG: Kyoto Encyclopedia of Genes and Genomes.

Given the strong immune-related enrichment signals, we investigated the relationship between hub genes and the tumor immune microenvironment. A heatmap illustrated distinct patterns of immune cell infiltration and stromal proportion between recurrence groups (Figure 6A). Correlation analysis revealed that KLF13 and TIA1 expression were significantly associated with the infiltration levels of specific immune cell types, KLF13 is correlated with T cells CD4 memory resting (r = 0.29, p = 0.07), and TIA1 is linked to Mast cells activated cells (r = 0.37, p = 0.02) (Figure 6B), cell types often associated with immunosuppression. Supporting this, KEGG and GO analyses of genes co-expressed with the recurrence phenotype highlighted profound involvement in immune response regulation and T cell activation pathways (Figures 6C,D). Further validation using the TIMER database across multiple cancer types (BRCA-basal, SKCM) confirmed that KLF13 and TIA1 expression levels were consistently correlated with the abundance of various tumor-infiltrating immune cells (Figures 6E,F).

### 3.7 Construction of a risk factor model and validation in internal data-set

**FIGURE 7:**
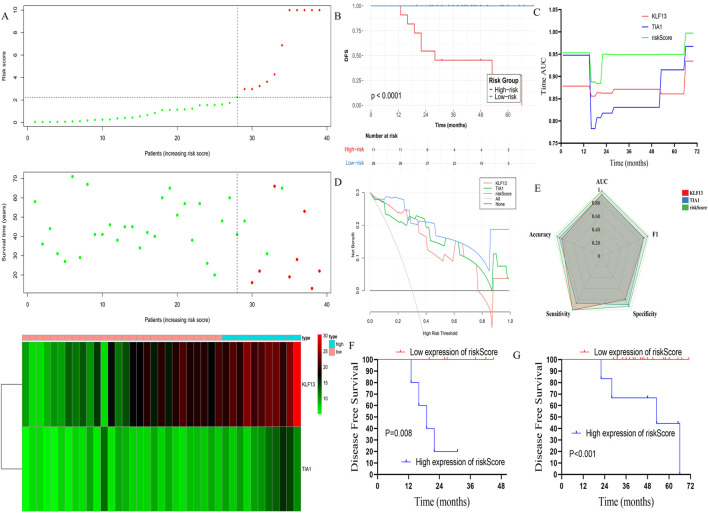
Rrisk factor model construction and verified in the prognosis in the internal dataset. **(A)** The risk factor model of the hub genes in the 39 MPD and EMPD patients. (Upper) Hub genes risk score distribution of 39 MPD and EMPD patients. (Middle) Status of every patient in the external dataset (N = 39). (Lower) Expression heatmap of the hub genes corresponding to each sample above. Red: high expression; Blue: low expression. **(B)** The DFS analysis of the risk score in the 39 MPD and EMPD patients. **(C)** Time-dependent AUC curves of the hub genes and risk factor model for the prediction of DFS in the 39 MPD and EMPD patients. **(D)** The DCA curves of the hub genes and risk factor model for recurrence in the internal dataset; **(E)** The radar chart shows the F1 score, accuracy score, sensitivity score, AUC value, and specificity score, for hub genes and risk factor model for recurrence in the internal dataset; **(F)** The K-M analysis of the risk factor model for the DFS in EMPD in internal dataset; **(G)** The K-M analysis of the risk factor model for the DFS in MPD in internal dataset. MPD: Mammary Paget’s disease, EMPD: extramammary Paget’s disease.K-M: Kaplan-Meier.

A Cox regression analysis performed to explore the disease free survival impact of the hub genes in PD patients demonstrated that the KLF13 (HR = 1.284, 95%CI: 1.080–1.527, P < 0.001) and TIA1 (HR = 1.463, 95%CI: 1.100–1.945, P < 0.001) were associated with the DFS. The risk score of the hub genes was calculated using the formulae: risk score = 0.25 × KLF13 expression +0.38 × TIA1 expression, based on the COX regression analysis. The patients were subsequently divided into the high and low-risk groups (Figure 7A). Notably, patients in the low-risk group had an improved DFS than those in the high-risk group (N = 39, log-rank P < 0.001, Figure 7B). Time-dependent receiver operating characteristic (ROC) analysis demonstrated that the risk-score model (AUC = 0.997) outperformed either hub gene alone in predicting 1-, 3-, and 5-year DFS (Figure 7C). Decision curve analysis (DCA) confirmed the superior clinical net benefit of the risk model over individual genes or traditional clinical factors (Figure 7D). A comprehensive radar chart evaluating multiple metrics (F1 score, accuracy, sensitivity, specificity, AUC) further illustrated the robust predictive performance of the integrated model (Figure 7E). The model’s prognostic power remained significant when applied to the EMPD and MPD subgroups separately (Figures 7F,G).

### 3.8 Validation of the risk score in the external data set

**FIGURE 8:**
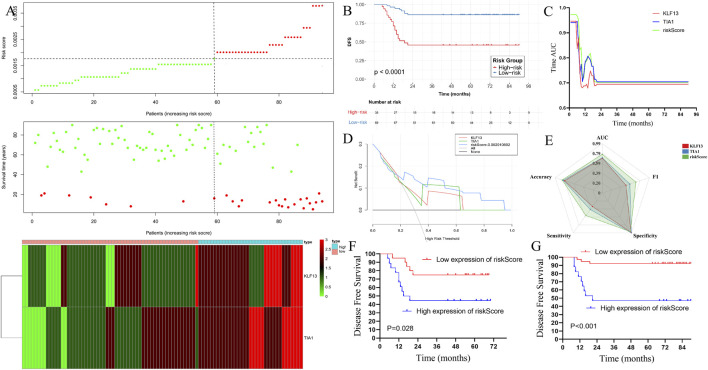
Rrisk factor model construction and verified in the prognosis in the externaldataset. **(A)** The risk factor model of the hub genes in the 94 MPD and EMPD patients. (Upper) Hub genes risk score distribution of 94 MPD and EMPD patients. (Middle) Status of every patient in the external dataset (N = 94). (Lower) Expression heatmap of the hub genes corresponding to each sample above. Red: high expression; Blue: low expression. **(B)** The DFS analysis of the risk score in the 94 MPD and EMPD patients. **(C)** Time-dependent AUC curves of the hub genes and risk factor model for the prediction of DFS in the 94 MPD and EMPD patients. **(D)** The DCA curves of the hub genes and risk factor model for recurrence in the external dataset; **(E)** The radar chart shows the F1 score, accuracy score, sensitivity score, AUC value, and specificity score, for hub genes and risk factor model for recurrence in the external dataset; **(F)** The K-M analysis of the risk factor model for the DFS in EMPD in external dataset; **(G)** The K-M analysis of the risk factor model for the DFS in MPD in external dataset. MPD: Mammary Paget’s disease, EMPD: extramammary Paget’s disease, AUC: Area under the curve, K-M: Kaplan-Meier. DCA: Decision curve analysis.

To further explored the risk score model can predict the prognosis in PD patients. Basing on the COX regression result, we calculated the risk score in each patients in external data set. Then, the patients were divided into two groups, high risk score group and low risk score group (Figure 8A). Notably, patients in the low-risk group had an improved DFS than those in the high-risk group (N = 94, log-rank P < 0.001, Figure 8B). Time-dependent ROC analysis demonstrated that the risk-score model (AUC = 0.702) outperformed either hub gene alone in predicting 1-, 3-, and 5-year DFS (Figure 8C). Decision curve analysis (DCA) confirmed the superior clinical net benefit of the risk model over individual genes or traditional clinical factors (Figure 8D). A comprehensive radar chart evaluating multiple metrics (F1 score, accuracy, sensitivity, specificity, AUC) further illustrated the robust predictive performance of the integrated model (Figure 8E). The model’s prognostic power remained significant when applied to the EMPD and MPD subgroups separately (Figures 8F,G).

### 3.9 Validation of hub gene expression in internal dataset and functional analysis

**FIGURE 9:**
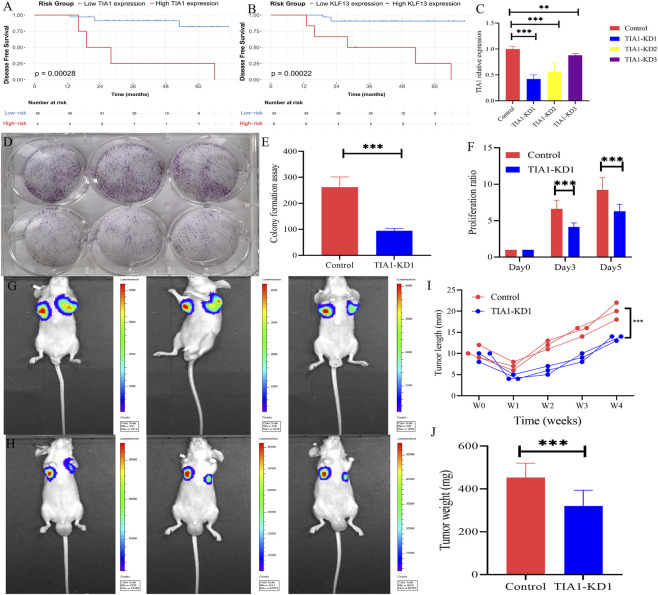
Validation of hub genes using qPCR, *in vitro* and *in vivo* experiments. **(A)** Kaplan-Meier analysis of TIA1 expression for disease-free survival (DFS) in all patients from the internal cohort; **(B)** Kaplan-Meier analysis of KLF13 expression for DFS in the external cohort; **(C)** Relative TIA1 expression in MDA-MB-231 cells following knockdown; **(D and E)** Colony formation assays showing significantly reduced colony numbers in TIA1-KD1 cells compared to parental controls (n = 3 per group). **(F)** CCK-8 assays demonstrating significantly decreased proliferation in TIA1-knockdown (TIA1-KD1) cells; Tumor growth was monitored in Control and TIA1-KD1 mice by bioluminescence imaging at week 1 **(G)** and week 4 **(H)** (n = 3 per group). **(I and J)** Tumor growth curves of MDA-MB-231 xenografts from the TIA1-KD1 and control groups. KD: knockdown. *P < 0.05, **P < 0.01, ***P < 0.001.

Kaplan-Meier analysis of the internal cohort demonstrated that TIA1 expression was significantly associated with DFS, with effective patient stratification by expression levels (Figure 9A). Consistent with these findings, KLF13 expression showed prognostic value in the external validation cohort (Figure 9B). To functionally characterize TIA1 in a breast cancer model analogous to MPD, we established lentiviral-mediated TIA1 knockdown in MDA-MB-231 cells. RT-qPCR validated efficient TIA1 silencing (Figure 9C), and the TIA1-KD1 group has the best knock down effeciency which was selected for all subsequent experiments. Subsequent functional analyses revealed that TIA1 depletion significantly impaired clonogenic potential (colony formation assay, Figures 9D,E) and cell viability (CCK-8 assay, Figure 9F) compared to parental controls. Moreover, tumor xenografts examined *in vivo* in the two groups to explore the function of TIA1 further confirmed TIA1 to be an oncogene (all P < 0.01; Figures 9G–J).

## 4 Discussions

PD, encompassing both mammary MPD and EMPD subtypes, is a malignant cutaneous neoplasm with distinct prognostic profiles. Notably, MPD is frequently associated with distant metastasis, while EMPD is characterized by a higher propensity for local recurrence. To elucidate the differential mechanisms underlying recurrence and to identify efficient biomarkers for precise recurrence prediction, we performed RNA sequencing on PD samples. Our analysis revealed that MPD recurrence signatures were closely linked to biological processes such as epidermis development, skin morphogenesis, and cornification. Conversely, EMPD recurrence was associated with a more prominent inflammatory and immune-modulatory tumor microenvironment. Using WGCNA, we identified KLF13 and TIA1 as hub genes. The prognostic significance of these genes was subsequently validated in both internal and external patient datasets. Finally, a risk score model based on these markers was constructed, effectively stratifying PD patients according to their clinical outcomes.

The diagnosis of PD relies heavily on histopathological and immunohistochemical analysis. MPD is distinguished by Paget cells within the nipple epidermis, frequently confirmed by markers such as cytokeratin 7 (CK7) and mucin 1 (MUC1), reflecting its breast cancer association (Liegl et al., 2007). EMPD, however, often expresses CK7 and mucin5AC (MUC5AC), the latter distinguishing it from MPD and highlighting its apocrine gland origin (Kuan et al., 2001; Kanitakis, 2007). In the previous study, there have several reports demonstrated that the pathophysiology of EMPD and MPD is driven by distinct molecular pathways and markers. The MPD was associated with the frequent overexpression and gene amplification of HER2 (Gatalica et al., 2020; Tanaka et al., 2013). In contrast, the PI3K-AKT-mTOR signaling pathway, marked by frequent activating mutations in EMPD (T et al., 2020; Chen et al., 2009). In the present study, our comparative transcriptomic analysis reinforces the growing consensus that MPD and EMPD, while histologically similar, are underpinned by distinct molecular landscapes. The identification of over 2,700 DEGs strongly supports their biological heterogeneity. For MPD, the pathways related to epidermis development, skin development, and cornification is particularly intriguing. In contrast, the enrichment patterns in EMPD recurrence point toward a more pronounced inflammatory and immune-modulatory milieu.

Although histologically indistinguishable by conventional microscopy, MPD and EMPD exhibit distinct clinical behaviors and management strategies. MPD typically metastasizes distally, whereas EMPD demonstrates predominantly local recurrence, a pattern confirmed in our cohort. These divergent natural histories necessitate different therapeutic approaches. Given overlapping histopathological features on hematoxylin-eosin staining, immunohistochemical markers have been extensively explored. Classical biomarkers (ER, PR, CEA, HER2) show inconsistent expression across both entities, limiting diagnostic specificity (Lloyd and Flanagan, 2000). Emerging data indicate that mucin profiles, particularly MUC5AC expression in EMPD (absent in MPD) and MUC2 negativity (present in MPD), may provide more reliable discrimination (Inokuchi and Sasai, 1992) Identifying biomarkers that reliably predict recurrence is therefore clinically pertinent. To move beyond the noise inherent in standard differential expression analysis, we applied WGCNA, which clusters genes into co-expression modules based on topological overlap, thereby isolating functionally coherent gene sets associated with recurrence (Zhang and Horvath, 2005; Langfelder et al., 2008; Tian et al., 2017). The cyan module emerged as the key recurrence-associated module. Enrichment analysis highlighted its involvement in transcriptional regulation and choline metabolism. Subsequent LASSO regression pinpointed KLF13 and TIA1 as central hub genes, supporting their role as predictive biomarkers for recurrence in PD.

Krüppel-like factor 13 (KLF13) exhibits context-dependent roles in tumorigenesis, functioning as an oncogene in cancers such as oral squamous cell carcinoma (Henson and Gollin, 2010), cervical cancer (Zhang et al., 2016), and esophageal cancer (Yang et al., 2025), while acting as a tumor suppressor in colorectal cancer (Yao et al., 2020), gastric cancer (Ding et al., 2022), glioma (Hu et al., 2019), and prostate cancer (Wang et al., 2018). In the present study, KLF13 demonstrated a pro-tumorigenic role in both MPD and EMPD, with its overexpression correlating with unfavorable prognosis. Furthermore, our immune correlation analysis revealed a novel association between high KLF13 expression and an immunosuppressive tumor microenvironment-a mechanistic insight not previously reported in KLF13-related oncology research. These findings position KLF13 not only as an intrinsic oncogenic driver in Paget’s disease but also as a potential regulator of the local immune landscape.

TIA1 (T-cell intracellular antigen 1) is an RNA-binding protein central to stress granule formation and post-transcriptional regulation (Tian et al., 1991; Kawakami et al., 1994; Izquierdo and Valcárcel, 2007). Its role in oncology is context-dependent, associated with both tumor-suppressive functions, in ovarian cancer (Milne et al., 2009) and urological cancer (Kijima et al., 2022), where its loss correlates with progression and oncogenic activities in breast cancer, colon cancer and hepatocellular cancer (Hong et al., 2020; Bakheet et al., 2025). Our study supports a pro-tumorigenic role for TIA1 in Paget’s disease, where elevated expression correlates with poor prognosis. Notably, we identified a novel association between high TIA1 levels and increased infiltration of immunosuppressive cells, suggesting a potential link between TIA1-mediated stress response and the establishment of an immune-tolerant tumor microenvironment in PD-a mechanistic hypothesis that warrants further experimental investigation.

Risk factor models have been widely reported in cancers such as melanoma, lung cancer, and rectal cancer (Zhang et al., 2021; Meng et al., 2022). In this study, based on RNA-seq data, we constructed a risk-score model that effectively predicted prognosis in PD patients. The model was further validated in external datasets. Time-dependent ROC analysis demonstrated that the combined model achieved a higher AUC value than either hub gene alone in predicting patient outcomes. Importantly, the risk score also exhibited strong prognostic performance in external validation cohorts. In summary, our risk-score model shows robust and generalizable ability to stratify PD patients by prognosis.

Despite these findings, several limitations should be acknowledged. First, the prognostic relevance of the identified hub genes was validated primarily via immunohistochemical analysis, warranting further confirmation in larger, independent cohorts. Second, the functional roles of KLF13 and TIA1 were inferred from bioinformatics analyses; mechanistic validation through *in vitro* and *in vivo* experiments is required to substantiate these observations. Thirdly, the sample size for EMPD patients was relatively small which may resulting to the overfitting. Therefore, larger cohorts from multiple centers should be analyzed. Fourth, we acknowledge that due to the current absence of MPD and EMPD cell lines, breast cancer cell lines were used as an alternative in this study. We hope that MPD/EMPD-specific cell lines can be established for validation in future experiments. Moreover, during the *in vivo* and *in vitro* validation, we only validated the TIA1 and did not include the KLF13, which may affect the robustness of our findings. Furthermore, in the Kaplan-Meier analyses where X-tile was employed to determine optimal cutoffs, we did not apply multiple-testing corrections (e.g., FDR or Bonferroni). Consequently, the possibility of inflated false-positive findings should be considered when interpreting these results. Future studies incorporating expanded sample sizes and functional assays will help strengthen the biological and clinical implications of our findings.

## 5 Conclusion

In summary, RNA-seq analysis revealed distinct functional profiles between MPD and EMPD, with recurrence in MPD associated with developmental and differentiation pathways, while EMPD recurrence was linked to immune and inflammatory processes. Using WGCNA, we identified KLF13 and TIA1 as hub genes, which were subsequently validated as independent prognostic biomarkers in internal and external datasets. A risk-score model based on these genes effectively stratified patients by outcome. Further studies are needed to elucidate the detailed molecular mechanisms underlying their roles in PD progression and recurrence.

## Data Availability

The original contributions presented in the study are publicly available. This data can be found in the GEO database with the accession number GSE328302.

## Supplementary material

SUPPELMENTARY FIGURE S1 The soft threshold of theWeighted gene co-expression network analysis (WGCNA).
