# Supplementary figures and images for: Identification of two genes associated with recurrence in Paget’s disease and construction of a predictive model
Source: Front Genet. 2026 May 13;17:1784429. doi: 10.3389/fgene.2026.1784429 (PMC13211854; doi:10.3389/fgene.2026.1784429)

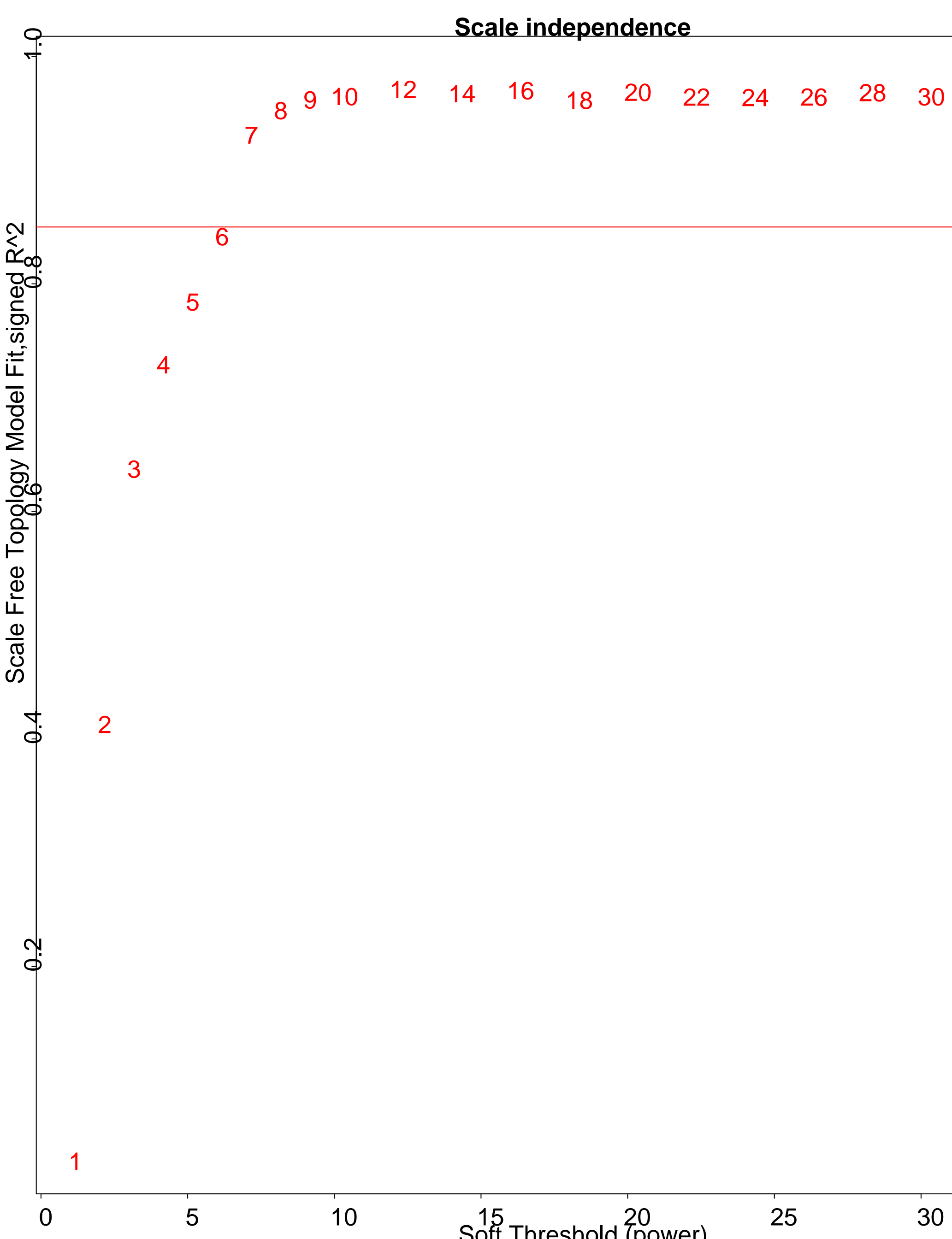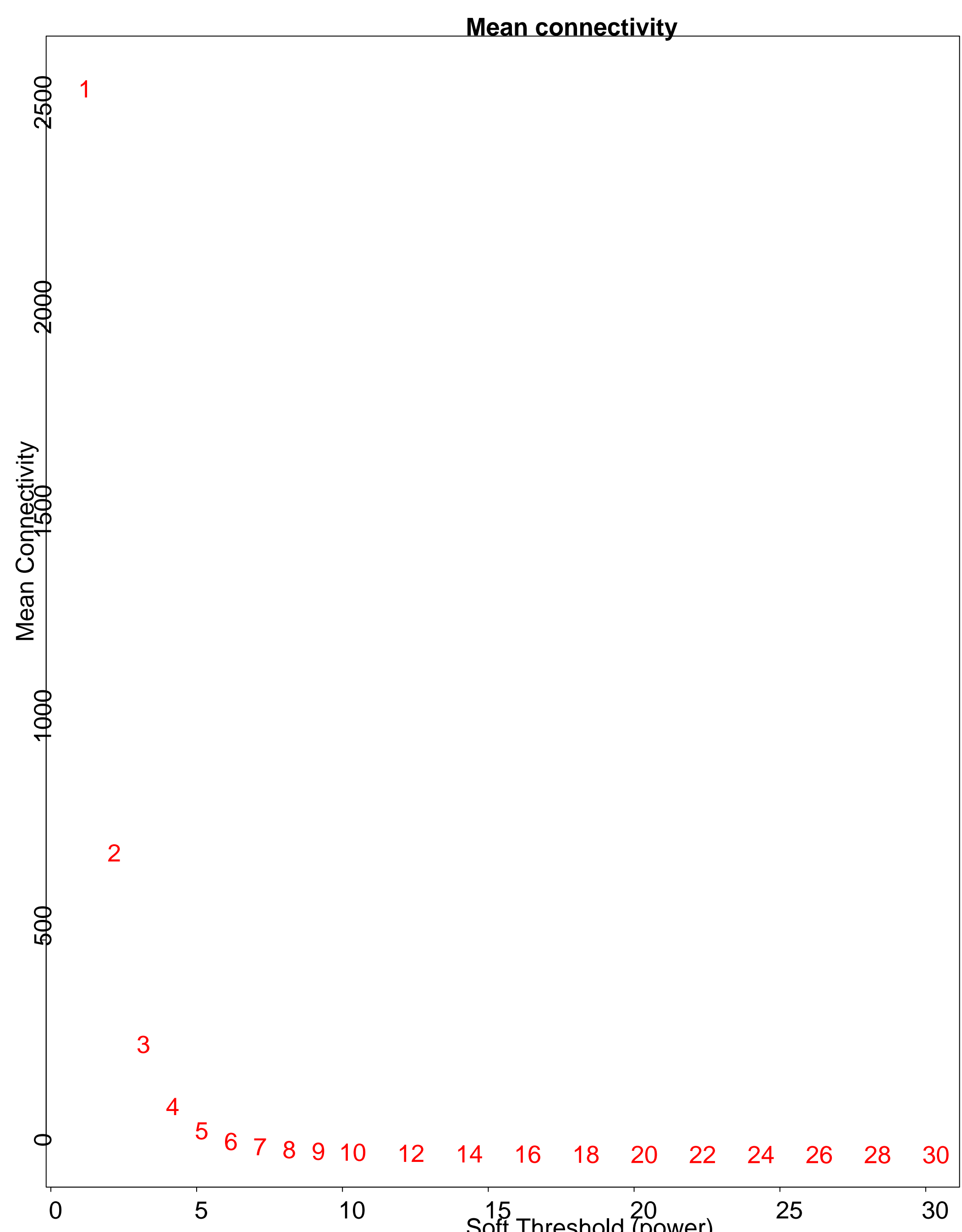

Supplement: Supplementary file 1 [file DataSheet2.pdf]
