# Supplementary material for: Identification of two genes associated with recurrence in Paget’s disease and construction of a predictive model
Source: Front Genet. 2026 May 13;17:1784429. doi: 10.3389/fgene.2026.1784429 (PMC13211854; doi:10.3389/fgene.2026.1784429)
Supplement: Supplementary file 2 [file Table1.docx]

Supplementary Table 1 Baseline characteristics of Paget’s disease patients in two dataset

| **Characteristics** | **RNA-seq(n=39)** | | **P-value** | **IHC data-set(n=94)** | | **P-value** |
| --- | --- | --- | --- | --- | --- | --- |
|  | **EMPD(n=11)** | **MPD(n=28)** |  | **EMPD(n=38)** | **MPD(n=56)** |  |
| Sex (%) |  |  | <0.001 |  |  | <0.001 |
| Male | 7.00 (63.6%) | 0 (0%) |  | 24.0 (63.2%) | 2.00 (3.6%) |  |
| Female | 4.00 (36.4%) | 28.0 (100%) |  | 14.0 (36.8%) | 54.0 (96.4%) |  |
| Age (years) | 63.5 ± 8.23 | 57.9 ± 9.29 | 0.238 | 61.8 ±10.6) | 58.2 ±10.4) | 0.269 |
| Tumor size | 4.21 ± 1.20 | 1.83± 0.879 | <0.001 | 3.08 ±1.08) | 2.44 ±1.02) | 0.0178 |
| Pathological T stage (%) |  |  | 0.744 |  |  | 0.00926 |
| 1 | 4.00 (36.4%) | 14.0 (50.0%) |  | 31.0 (81.6%) | 25.0 (44.6%) |  |
| 2 | 7.00 (63.6%) | 14.0 (50.0%) |  | 7.00 (18.4%) | 27.0 (48.2%) |  |
| 3 | 0 (0%) | 0 (0%) |  | 0 (0%) | 4.00 (7.1%) |  |
| Pathological N stage (%) |  |  | 0.566 |  |  | 0.795 |
| N0 | 8.00 (72.7%) | 12.0 (42.9%) |  | 28.0 (73.7%) | 37.0 (66.1%) |  |
| N1 | 3.00 (27.3%) | 15.0 (53.6%) |  | 10.0 (26.3%) | 17.0 (30.4%) |  |
| N2 | 0 (0%) | 1.00 (3.6%) |  | 0 (0%) | 2.00 (3.6%) |  |
| Pathological TNM stage (%) |  |  | 0.214 |  |  | 0.0343 |
| 1 | 4.00 (36.4%) | 8.00 (28.6%) |  | 25.0 (65.8%) | 20.0 (35.7%) |  |
| 2 | 4.00 (36.4%) | 19.0 (67.9%) |  | 9.00 (23.7%) | 32.0 (57.1%) |  |
| 3 | 3.00 (27.3%) | 1.00 (3.6%) |  | 4.00 (10.5%) | 4.00 (7.1%) |  |
| Recurrence | 4.00 (36.4%) | 4.00 (14.3%) | 0.307 | 15.0 (39.5%) | 12.0 (21.4%) | 0.165 |
