# Supplementary material for: Identification of two genes associated with recurrence in Paget’s disease and construction of a predictive model
Source: Front Genet. 2026 May 13;17:1784429. doi: 10.3389/fgene.2026.1784429 (PMC13211854; doi:10.3389/fgene.2026.1784429)
Supplement: Supplementary file 3 [file Table2.docx]

Table S2 Sequences of Primers for qPCR

| Gene | F | R |
| --- | --- | --- |
| KLF13 | CCTAGCGGACCTCAACCAGCAA | GCGTAGTGGCACTTGTGCTTCC |
| TIA1 | ATTTCCATGTCTTTGTTGGT | GATATTCTTCCAAATGGTG |
| GAPDH | GGGAAACTGTGGCGTGAT | GAGTGGGTGTCGCTGTTGA |
